# Phosphate-Surface-Modified Silica Nanoparticles for 5-Fluorouracil as a Prolonged Drug Delivery System

**DOI:** 10.3390/ph19050802

**Published:** 2026-05-21

**Authors:** Aleksandra Lis, Arkadiusz Surażyński, Przemysław Koźmiński, Paweł Szymański

**Affiliations:** 1 Department of Pharmaceutical Chemistry, Drug Analyses and Radiopharmacy, Faculty of Pharmacy, Medical University of Lodz, Muszynskiego 1, 90-151 Lodz, Poland; aleksandra.lis@umed.lodz.pl; 2 Department of Medicinal Chemistry, Faculty of Pharmacy with the Division of Laboratory Medicine, Medical University of Bialystok, Mickiewicza 2d, 15-222 Bialystok, Poland; arkadiusz.surazynski@umb.edu.pl; 3 Centre of Radiochemistry and Nuclear Chemistry, Institute of Nuclear Chemistry and Technology, Dorodna 16, 03-195 Warsaw, Poland; p.kozminski@ichtj.waw.pl; 4 Department of Biological Medicine, Military Institute of Hygiene and Epidemiology, Kozielska 4, 01-163 Warsaw, Poland

**Keywords:** anticancer therapy, prolonged drug release, silica nanoparticles, targeted therapy, 5-fluorouracil

## Abstract

**Background/Objectives:** This paper describes the synthesis of silica nanoparticles (SiNPs) and their surface modification with amino and phosphate groups (SiNPs-NH_2_-PO_3_). The functionalized nanoparticles were subsequently loaded with the anticancer drug 5-fluorouracil (SiNPs-NH_2_-PO_3_-5-FLU) and further modified with PEG2000 (SiNPs-NH_2_-PO_3_-5-FLU-PEG2000). **Methods:** In this study, a one-step, two-phase, sol–gel method carried out at room temperature was used to synthesize the nanoparticles. The size and surface zeta potential of the created SiNPs were determined by DLS measurements. HPLC was used to determine the amount of drug loaded into silica nanoparticles and the drug release profile in two different pH environments (slightly acidic and physiological). Based on physicochemical characteristics, the SiNPs-NH_2_-PO_3_-5-FLU and SiNPs-NH_2_-PO_3_-5-FLU-PEG2000 formulations were chosen for comprehensive characterization. The cytotoxicity of the studied complexes was assessed in MCF7 breast cancer cells, while their ability to induce apoptosis in those cells was examined using specific immunofluorescence markers: active caspase-7, active poly(ADP-ribose) polymerase (PARP), and p53 protein. **Results:** Our findings demonstrate that SiNPs-NH_2_-PO_3_-5-FLU can induce a stronger apoptotic response than free 5-FLU at equivalent concentrations. We observed that drug release occurs not only under physiological conditions but is further enhanced in a mildly acidic environment (pH 5.0), characteristic of the tumor microenvironment. **Conclusions:** Most 5-fluorouracil formulations are administered as injectable solutions, resulting in systemic exposure and significant adverse effects. However, their encapsulation within nanoparticles could favor preferential drug release in the acidic tumor microenvironment, thus supporting targeted therapy and reducing toxicity to healthy tissues. Moreover, PEGylation of the nanoformulation allows prolonged and controlled release.

## 1. Introduction

The term nanotechnology was coined by the Nobel laureate Richard Feynman in his 1959 American Physical Society lecture ‘‘There’s Plenty of Room at the Bottom” [1]. Nanotechnology is a scientific discipline concerned with the properties of materials at the nanoscale, especially solid-state materials [2], which may demonstrate different surface effects and quantum effects than other materials on the larger scale [3]. The International Organization for Standardization (ISO) defines nanoparticles (NPs) as nano-objects whose external dimensions all fall within the nanoscale, with the lengths of the longest and shortest axes being relatively similar [4].

Nanosystems are characterized by variable sizes: nanocrystals and quantum dots range from 2 to 9.5 nm, while dendrimers are usually up to 10 nm, polymeric micelles from 10 to 100 nm, metallic nanoparticles from 1 to 100 nm, liposomes from 50 to 100 nm, and polymeric nanoparticles from 10 to even 1000 nm [5,6]. When in an ionic solution, NPs with a net surface charge are surrounded by a Stern layer of oppositely charged ions that are firmly attached to the particle, which are in turn surrounded by a more loosely associated electrical double layer of ions. As the NPs are moved around under the influence of Brownian diffusion or applied force, some ions in the diffuse layer move with the NP while others remain in the surrounding bulk solution. This causes the formation of a “slipping plane” boundary, referred to as the zeta potential, whose electrostatic potential is intrinsically linked to the surface charge of the NPs [7]. The zeta potential is of particular significance in characterizing drug delivery from mucous membranes, such as those from the nasal, ocular, vaginal, or gastrointestinal area [8]. Mucous sites possess anionic compounds, such as sialic acids, which attract positively charged nanoparticles and immobilize them while allowing negatively charged or uncharged NPs to pass through the mucus [9]. The surface charge of NPs also influences drug loading capacity [10].

Recent years have seen continual growth in the incidence and mortality of cancer, which presents an ongoing challenge for researchers [11]. Most treatment is currently based on surgery, radiotherapy, and chemotherapy [12], with chemotherapy being the most commonly available approach for cancer treatment. Unfortunately, many chemotherapeutic drugs do not reach the target site and can cause numerous adverse effects and drug resistance [13]. There is hence a need for new drug delivery systems (DDSs) that can improve drug selectivity and enable combination therapy.

One such approach involves the use of NPs loaded with drugs, which has been broadly studied as a means of drug delivery [14]. While conventional dosage forms are characterized by low bioavailability and efficacy, systemic adverse effects, and degradation in the digestive system [15], NP-based DDSs allow precise targeting of tumor sites and prolonged half-life in circulation [16].

**Figure 1:**
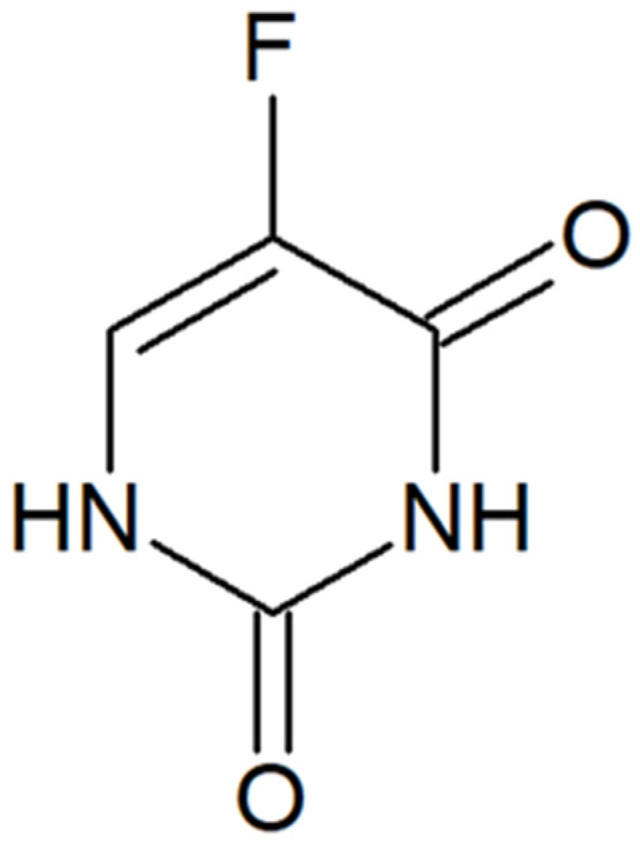
The chemical structure of 5-fluorouracil.

In recent years, several nanomedicines based around liposomes, nanocrystals, PEGylated polymeric nanoparticles, and metal or protein-based nanoparticles have been approved for use by the FDA; for example, Spikevax (ModernaTX Inc., Cambridge, MA, USA) is a nucleoside-modified mRNA (vaccine) loaded into lipid nanoparticles used in the treatment of coronavirus disease 2019 (COVID-19) [17]. Another drug, known since the 1950s, is 5-fluorouracil (5-FLU) [18], Figure 1, used for various neoplasms, including head and neck squamous cell carcinoma (SCC) [19], gastrointestinal SCC and adenocarcinoma (ADC) [20,21], and SCC of the uterine cervix [22]. After application, 5-FLU exerts its cytotoxic properties by inhibiting cellular thymidylate synthase (TS), thus preventing DNA replication [23], and inhibiting RNA synthesis by integration into RNA [24].

One of the most common neoplasms of the digestive system, with high morbidity and mortality, is colorectal cancer [25]. Unfortunately, 5-FLU typically demonstrates poor absorption in the gastrointestinal tract when administered orally; as such, it is usually systemically administered, like other anticancer drugs. As a result, the entire body and all its organs are exposed to the toxic effects of the drug. 5-FLU can be administered via continuous intravenous infusion over several days, intravenous bolus, or infusion via ambulatory pump for one to two weeks [26]. After intravenous administration, the drug is subject to rapid catabolism in the liver, resulting in a terminal half-life of around 8 to 20 min [26].

The unique physicochemical and biological properties of nanoparticles make them suitable as novel drug delivery systems (DDSs) offering improved pharmacokinetic properties and enhanced therapeutic efficacy. Additionally, these DDSs improve the pharmacodynamic properties of the encapsulated drugs, facilitating targeted drug delivery, greater stability, or controlled drug release [27]. The field of drug delivery has gained significant attention in pharmaceutical and medical sciences as a means to enhance therapeutic efficacy. The key aim of research into DDSs is to optimize the effectiveness of therapy while reducing the risk associated with adverse effects and enhancing patient adherence [28].

A key aspect of nanoparticles that makes them valuable candidates for DDSs is their ability to facilitate precise and gradual release of active substances from NPS over an extended period, resulting in better therapeutic effects [29]. Among these, silica-based nanoparticles offer good biocompatibility, a large surface area, and adjustable pore size (ranging from 2 to 50 nm, making them especially suitable for DDS. Hence, they have been widely used in pharmaceuticals, especially for the delivery of chemotherapeutics [30,31,32]. The drug molecules can be trapped in the NP mesopores through electrostatic interactions and subsequently released upon stimulation [33].

The present study describes the design and synthesis of polyethylene glycol (PEG 2000)-conjugated silica nanoplatforms loaded with 5-fluorouracil. The surface was further functionalized with amine and phosphate groups. While silica-based and PEGylated systems loaded with 5-FLU have been previously reported [34,35], the combined effect of phosphate groups and PEG chains on drug loading, surface charge modulation, and release behavior has not been systematically explored. PEG, also known as macrogol, is a linear polymer with unique hydrophilicity and electrical neutrality: thanks to its active hydroxyl groups at both ends, it easily binds to other groups on the surface of the NPs, creating a hydrophilic protective layer around the NP surface. This treatment significantly increases the half-life of the drug in circulation by reducing clearance through steric repulsion [36], which also provides colloidal stability and reduces NP aggregation [37].

## 2. Results

### 2.1. Synthesis of SiNPs and Surface-Modified Silica Nanoparticles

Silica nanoparticles (SiNPs) were modified with amine and phosphate groups (SiNPs-NH_2_-PO_3_) and loaded with the cytostatic drug 5-fluorouracil to form SiNPs-NH_2_-PO_3_-5-FLU. The surface of the loaded nanoparticles was also enriched with ligand PEG 2000 (SiNPs-NH_2_-PO_3_-5-FLU-PEG2000).

Silica was selected as a material for DDS formulation due to its unique properties, such as its outstanding compatibility with biological systems, easily modified surface area, and strong ability to encapsulate different compounds [38]. The major limitation in the synthesis of NPs is their tendency to aggregate due to the large surface area of the particles and the strong attraction between them [39]. As this propensity for aggregation can increase nanoparticle toxicity [40], the basic catalyst triethanolamine (TEA) was added to the formulation to prevent agglomeration and NP growth [41]. Cetyltrimethylammonium chloride (CTAC) was used as a cationic surfactant; the compound self-assembles into a micelle with a hydrophobic core, although the surfactant concentration has to be above the critical micelle concentration [42]. SiNP structures were generated by reacting cationic micelles with the anionic silica precursor tetraethyl orthosilicate (TEOS). n-hexane was used as a pore expander [43]. Following CTAC treatment, the SiNPs were subjected to calcination to remove any residual CTAC which may block the pores. Surfactant removal was confirmed by the lack of a surfactant peak at 2980 and 2930 cm^−1^ FTIR spectra post-calcination.

The SiNPs’ surface was modified by post-synthesis functionalization to alter its charge; this can influence the penetration of NPs into desired cells [44] or the rate of drug release [45]. Briefly, amine groups were first attached with organosilane APTES to form SiNPs-NH_2_, and then phosphate groups were added using THMP, resulting in the creation of SiNPs-NH_2_-PO_3_. One potential disadvantage regarding the use of SiNPs in biological applications such as DDSs is that they may adhere to proteins or blood platelets. Since it is due to hydrophobic interactions [46], hydrophilic PEG is added to improve the movement of the NPs in aqueous solutions and reduce the risk of protein adsorption or cell adhesion [47]. PEG surface modification may prevent many of the medically undesirable effects of NP use, and PEG-grafted NPs are used in pharmaceutical preparations, magnetic resonance imaging (MRI), and blood-compatible biomedical applications [48]. Furthermore, PEG modification also enhances colloidal stability and sustains drug release [49].

### 2.2. Scanning Electron Microscopy (SEM)

**Figure 2:**
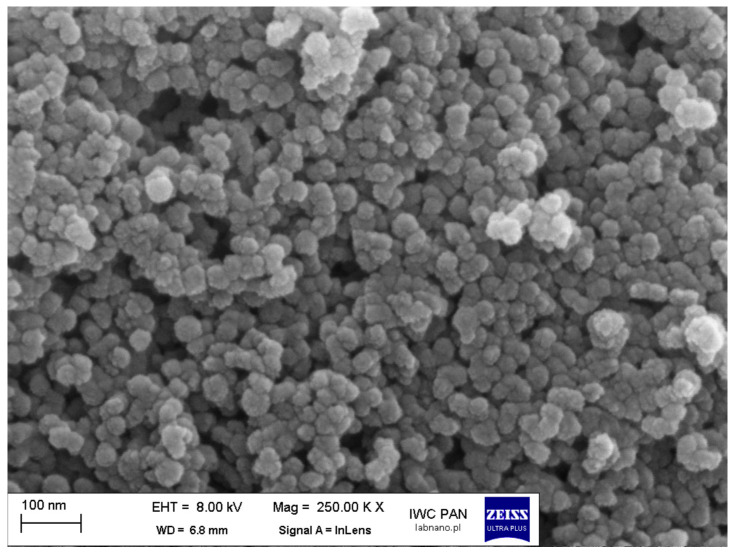
Scanning electron microscopy image: SiNPs-NH_2_-PO_3_-5-FLU-PEG2000, magnification 250.00 K X.

At lower magnification (25.00 K X), the SEM images (see Figure 2) revealed a sponge-like morphology formed by interconnected nanoparticle aggregates, indicating the presence of a three-dimensional network structure. At higher magnification (250.00 K X), the individual nanoparticles became clearly visible, exhibiting a predominantly spherical shape with a relatively uniform size distribution (~40 nm). In addition to individual particles, the presence of loosely bound aggregates was observed.

### 2.3. Dynamic Light Scattering (DLS)

The size of the created nanoparticles and their surface charge before and after surface modifications were determined by DLS studies (Table 1).

SiNPs, SiNPs-NH_2_, and SiNPs-NH_2_-PO_3_ were synthesized according to Janjua et al. [42]. The 5-FLU was loaded into SiNPs-NH_2_-PO_3_ on ice.

The SiNPs-NH_2_ and SiNPs-NH_2_-PO_3_ were found to be larger than the drug-loaded nanoparticles. The 5-FLU-loaded SiNPs-NH_2_-PO_3_ were 271.7 nm in diameter with −13.5 zeta potential. The introduction of PEG chains resulted in an increase in size and a decrease in absolute zeta potential.

### 2.4. Braunauer–Emmet–Teller (BET), Barrett–Joyner–Halenda (BJH), and Horvath–Kawazoe (HK) Pore Size Analysis

The BET analysis (see Table 2) revealed a high specific surface area of the material, amounting to 788 m^2^/g for nanoparticles with an unmodified surface. The high BET specific surface area value indicates a highly developed porous structure of the material. After surface modification, the specific surface area of the material decreased to approximately 300 m^2^/g, indicating a partial reduction in the accessibility of the porous structure to the adsorbate. The pore size distribution determined using the Horváth–Kawazoe method indicated a predominance of micropores with a diameter of approximately 0.8 nm. Due to the predominance of micropores, the Horváth–Kawazoe analysis provides more representative information about the porous structure than the Barrett–Joyner–Halenda (BJH) method, which is primarily intended for characterizing mesopores and widely used in nanoparticle research. According to International Union for Pure and Applied Chemistry (IUPAC) classification, the pores could be divided into three groups: micro (<2 nm), meso (2–50 nm), and macro (>50 nm) [50].

### 2.5. Fourier Transform Infrared Spectroscopy (FTIR)

The chemical structure of the samples was studied by Fourier transform infrared spectroscopy (FTIR) using a Spectrum Two FT-IR Spectrometer (Perkin Elmer, Waltham, MA, USA) in the range of 4000–400 cm^−1^.

**Figure 3:**
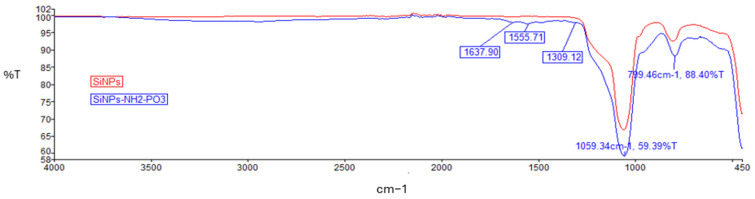
FTIR spectrum of SiNPs and SiNPs-NH_2_-PO_3_ with characteristic peaks marked in blue.

**Figure 4:**
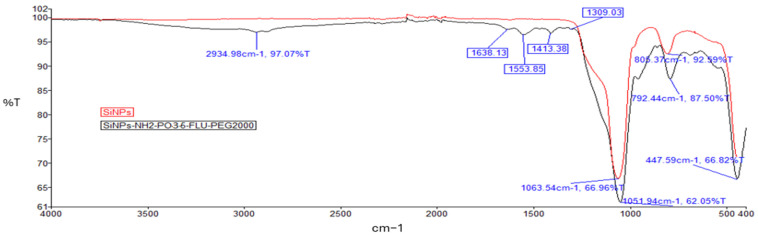
FTIR spectrum of SiNPs and SiNPs-NH_2_-PO_3_-5-FLU-PEG2000 with characteristic peaks marked in blue.

Successful modifications of the nanoparticles were confirmed using FTIR. An absorption peak was noted at 1100–1000 cm^−1^, which represents Si–O–Si asymmetric stretching vibration, and at 799.46 (Figure 3) or 792.44 and 805.37 cm^−1^ (Figure 4), indicating asymmetric bending and stretching vibration of Si–OH [51]. Unlike pure silica nanoparticles, the FTIR spectra of the final products: SiNPs-NH_2_-PO_3_-5-FLU and SiNPs-NH_2_-PO_3_-5-FLU-PEG2000 were found to have peaks at ~1309 cm^−1^ from the free phosphate groups [52]. The appearance of the ν(P=O) vibration in the 1100–1000 cm^−1^ region overlaps with the very intense Si–O–Si band, resulting in broadening and the formation of a shoulder at the main band centered at 1063 cm^−1^ (Figure 4) [53], whereas peaks at 1555.71 cm^−1^ and 1637.90 cm^−1^ (Figure 3) as well as 1553.85 cm^−1^ and 1638.13 cm^−1^ (Figure 4) correspond to amine groups [54]. Additionally, new absorption peaks at 1413.38 cm^−1^ and 2934.98 cm^−1^ (Figure 4) were noted, corresponding to the stretching vibration of alkyl groups from PEG2000 chains. A broad peak between 3600 and 3100 cm^−1^ (Figure 4) was observed, characteristic of the presence of hydroxyl groups from the PEG2000 chain. Hence, the FTIR spectra confirmed the successful introduction of amine and phosphate groups and the PEG2000 chains onto the silica nanoparticles.

### 2.6. HPLC—Calibration Curve

**Figure 5:**
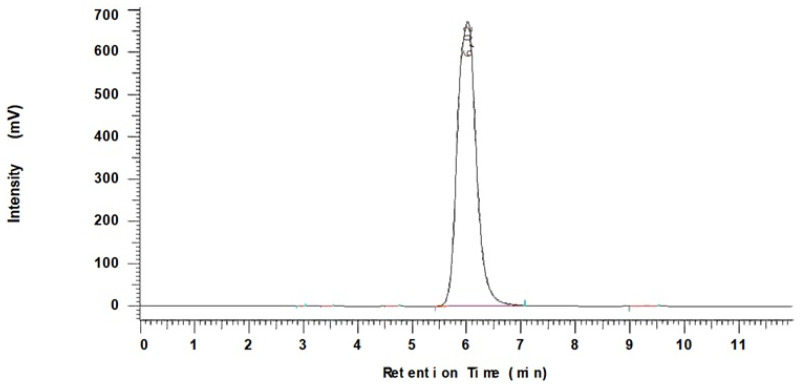
Representative HPLC chromatogram of 5-FLU was observed at a retention time of 6.02 min. The conditions were as follows: mobile phase: 1.5 mM K3PO4 buffer: ACN (95:5 *v*/*v*); flow rate 1 mL/min; UV detection wavelength: 210 nm; injection volume 10 μL; column temperature 25 °C.

The relationship between the amount of encapsulated drug and peak intensity was evaluated using a curve based on a certified 5-FLU standard. The determination coefficient (R^2^) of the calibration curve for 5-FLU was 0.9946. Figure 5 shows the peak of the 5-fluorouracil standard with a retention time of 6.02 min.

### 2.7. Quantification of Drug Loading

The amount of 5-FLU loaded into the SiNPs-NH_2_-PO_3_ particles was quantified indirectly by determining the residual amount of drug in the loading solutions following centrifugation of the dispersions (supernatant) [55]. The measurement was performed by high-performance liquid chromatography (HPLC).

Drug loading was determined based on mass balance using the following formula [56]:

(1) $$Drug\;loaded\;\left[mg\right]=Wstart-Wresidual$$

where *Drug Loaded* is the amount of 5-FLU confined in the SiNPs-NH_2_-PO_3_ in mg, *Wstart* is the starting amount of 5-FLU in the respective loading solution in mg, and *Wresidual* is the residual amount of 5-FLU in the supernatant in mg.

*Encapsulation efficiency* (*EE*) was calculated according to Equation (2):

(2) $$Encapsulation\;Efficieny\;\left(\%\right)=100\times \;\frac{WDrugLoaded}{Wstart}$$

where *WDrugLoaded* is the amount of 5-FLU confined in the SiNPs-NH_2_-PO_3_ in mg, as calculated using Equation (1). *Wstart* represents the starting amount of 5-FLU in the respective loading solution in mg.

Drug loading (DL) was calculated according to Equation (3):

(3) $$Drug\;Loading\;\left(\%\right)=100\times \;\frac{WDrugLoaded}{WDrugLoaded+WNPs}$$

where *WDrugLoaded* is the amount of 5-FLU confined in the SiNPs-NH_2_-PO_3_ in mg, as calculated using Equation (1). *WNPs* represent the total amount of nanoparticles (SiNPs-NH_2_-PO_3_) in the respective loading solution in [mg].

Finally, the *Theoretical Drug Loading* (*TDL*) was calculated according to the following formula:

(4) $$Theoretical\;Drug\;Loading\;\left(\%\right)=100\times \;\frac{Wstart}{Wstart+WNPs}$$

where *Wstart* is the starting amount of 5-FLU in the respective loading solution in mg, and *WNPs* represents the total amount of nanoparticles (SiNPs-NH_2_-PO_3_) in the respective loading solution in mg. “Based on the above equations, the parameters were calculated and collected in Table 3.

### 2.8. HPLC—In Vitro Release Studies

**Figure 6:**
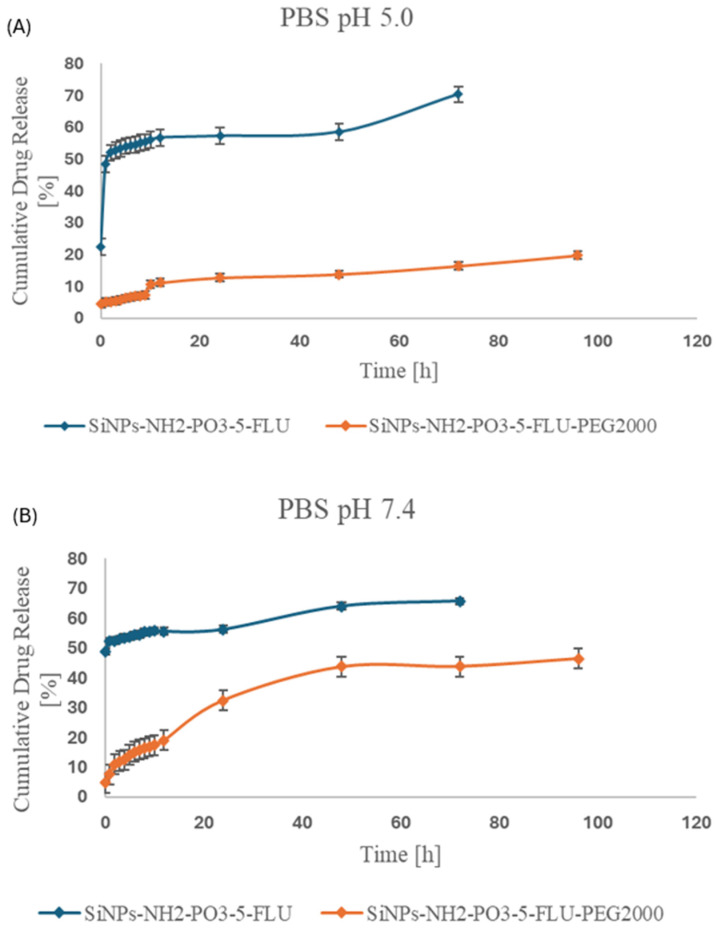
In vitro drug release study. The in vitro release of 5-FLU from SiNPs-NH_2_-PO_3_-5-FLU and SiNPs-NH_2_-PO_3_-5-FLU-PEG2000 was measured in phosphate-buffered solution at (**A**) pH 5.0 and (**B**) pH 7.4. The amount of 5-FLU was measured by HPLC; detection wavelength 210 nm. Data are shown as mean ± SEM (*n* = 3 independent experiments). Values of *p* ≤ 0.05 were considered statistically significant (one-way ANOVA).

The in vitro release of 5-FLU from SiNPs-NH_2_-PO_3_-5-FLU and SiNPs-NH_2_-PO_3_-5-FLU-PEG2000 was monitored in PBS solutions at two pH levels, 7.4 and pH 5.0 (Figure 6). It was found that 5-FLU was released from SiNPs-NH_2_-PO_3_-5-FLU at similar rates at pH 5.0 and pH 7.4. The release kinetics indicate rapid release of approximately 50% of the drug within the first five minutes, followed by this rate slowing, with 70% release recorded after 72 h.

The attachment of a PEG chain slowed drug release at both pH values, but the reduction was significantly more noticeable at pH 5.0. At PBS pH 7.4, approximately 20% of the drug was released within the first 12 h, around 30% after 24 h, and 40% at 48 h. After 72 and 96 h, an additional ~5% of the drug was released.

### 2.9. In Vitro Cytotoxicity and Apoptosis Tests

**Figure 7:**
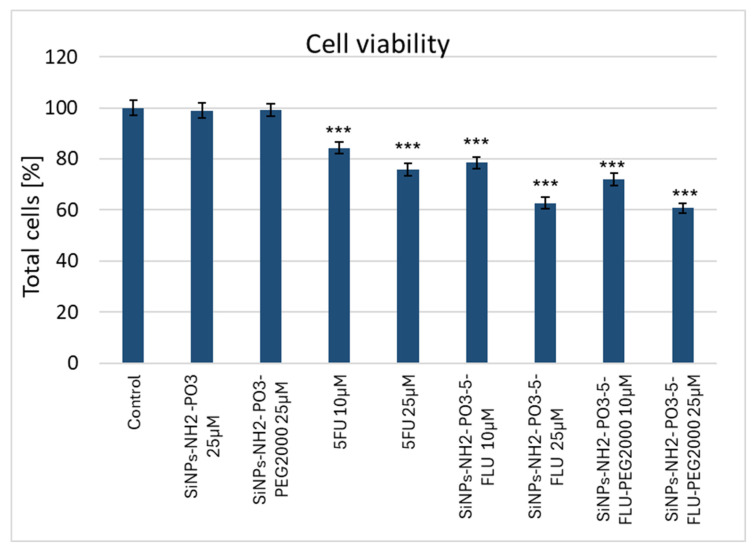
Effect of free 5-FLU, SiNPs-NH_2_-PO_3_-5-FLU, and SiNPs-NH_2_-PO_3_-5-FLU-PEG2000 on MCF7 cell viability after 48 h incubation at concentrations of 10 μM and 25 μM. Cell viability was determined using the MTT assay and expressed as percentage of untreated control cells. Data are presented as mean ± SD (*n* = 3 independent experiments). Statistical analysis was performed using one-way ANOVA. Statistical significance: *** *p* < 0.001 versus control.

**Figure 8:**
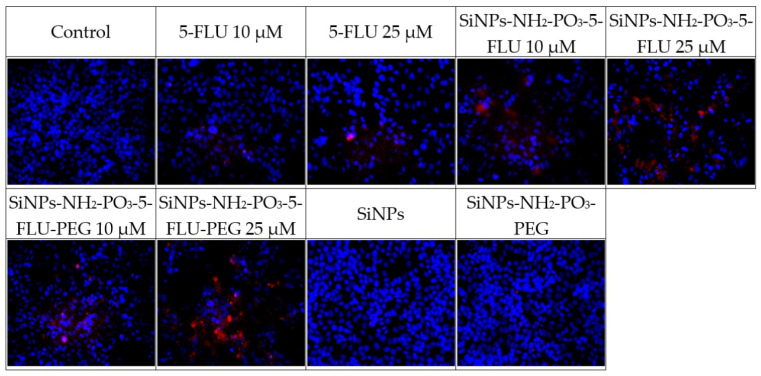
Expression of the active form of caspase 7 after 48 h of incubation with the tested substances at 10 μM and 25 μM. Values of *p* ≤ 0.05 were considered statistically significant (one-way ANOVA).

**Figure 9:**
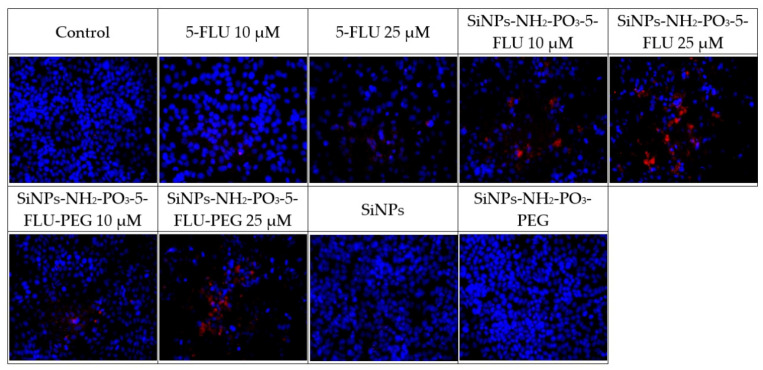
Expression of the active form of PARP after 48 h of incubation with the tested substances at 10 μM and 25 μM. Values of *p* ≤ 0.05 were considered statistically significant (one-way ANOVA).

**Figure 10:**
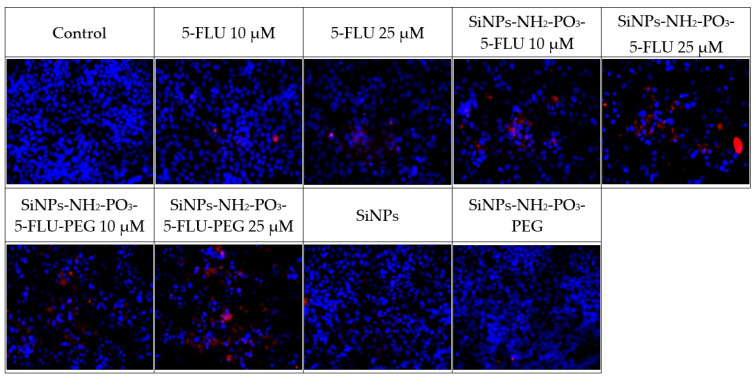
Expression of the active form of P53 after 48 h of incubation with the tested substances at 10 μM and 25 μM. Values of *p* ≤ 0.05 were considered statistically significant (one-way ANOVA).

These tests compared the effect of 5-FLU with its nanoparticle conjugates on MCF-7 breast cancer cells to assess whether the drug–carrier complex induces cytotoxicity (see Figure 7) and apoptosis (see Figure 8, Figure 9 and Figure 10). After 48 h, cell survival and the expression of apoptosis markers, viz. active caspase-7, active PARP, and p53 protein, were assessed in comparison to drug-free controls. The percentage of surviving cells and apoptosis marker levels were assessed based on fluorescence intensity in each of the tested 5-FLU combinations. Values of *p* ≤ 0.001 were considered statistically significant (one-way ANOVA).

#### 2.9.1. MTT Cell Viability Test

**Cell survival after 48 h of treatment with different concentrations of 5-FLU (10 µM and 25 µM)**

After 48 h of exposure to 5-FLU, a clear concentration-dependent cytotoxic effect was observed. At 10 µM 5-FLU, a moderate cytostatic effect was observed, with approximately 80% of MCF7 cells remaining alive compared to control cells. However, at 25 µM, the survival rate fell to about 50%. Importantly, 25 µM of 5-FLU had more than twice the cytotoxic effect of a lower concentration of 10 µM. Survival was assumed to be 100% in the untreated control sample.

**Cell survival after 48 h of treatment with SiNPs-NH_2_-PO_3_-5-FLU (10 µM and 25 µM)**

As in the case of the free drug, SiNPs-NH_2_-PO_3_-5-FLU also reduced cell survival in a concentration-dependent manner; however, the effects were noticeably higher than those of 5-FLU even at lower concentrations. After 48 h of exposure to 10 µM of SiNPs-NH_2_-PO_3_-5-FLU, cell survival fell to approximately 70% of control values (5-FLU showed no cytotoxic effect). At 25 µM, approximately 40% of the cells remained compared to controls. At 25 µM, the SiNPs-NH_2_-PO_3_-5-FLU had a more pronounced effect than the 25 µM of free 5-FLU, suggesting that binding 5-FLU to the nanocarrier increases its efficacy or availability to cells.

**Cell survival after 48 h of treatment with SiNPs-NH_2_-PO_3_-5-FLU-PEG2000**

The PEG-modified NP form, SiNPs-NH_2_-PO_3_-5-FLU-PEG2000, also demonstrated a concentration-dependent cytotoxic effect on MCF7 cells, although with a different intensity. After 48 h of exposure at 10 µM, cell survival was approximately 75–80% of control values (similar to the effect of 10 µM of free 5-FLU). At 25 µM, cell survival fell to approximately 50% of control values, indicating a strong cytotoxic effect comparable to that of free 5-FLU. Hence, SiNPs-NH_2_-PO_3_-5-FLU-PEG2000 retain the efficacy of 5-FLU; however, at 10 µM, this effect was comparable to that of free 5-FLU, i.e., slightly weaker than the non-PEG NPs.

#### 2.9.2. Immunofluorescence Analysis

All tested forms of 5-FLU, viz. classic free 5-FLU, SiNPs-NH_2_-PO_3_-5-FLU, and SiNPs-NH_2_-PO_3_-5-FLU-PEG2000, induce apoptosis in MCF7 cells, with the effect being dependent on concentration. This effect is evidenced by increased caspase-7 activity and PARP cleavage, accumulation of p53 protein in cell nuclei, and reduced cell survival. All cases demonstrated activation of intracellular pathways leading to programmed cell death.

**Expression of apoptosis markers after 48 h of treatment with different concentrations of 5-FLU (active caspase-7, PARP, p53)**

Increasing doses of 5-FLU correlated with apoptosis marker expression and thus, lower survival. In control cells, i.e., those without the drug, virtually no red fluorescence was detected for caspase-7, PARP, or p53. After 48 h of incubation with 5-FLU, strong expression of active caspase-7 and cleaved PARP was noted, indicating the activation of apoptosis. The expression of p53 protein also increased significantly, reflected in its accumulation in the cell nucleus, indicating a cellular response to DNA damage caused by 5-FLU.

**Expression of apoptosis markers after 48 h of treatment with SiNPs-NH_2_-PO_3_-5-FLU**

After 48 h of incubation with SiNPs-NH_2_-PO_3_-5-FLU, the expression of apoptotic markers was also observed. Treatment with these 5-FLU NPs induced strong expression of active caspase-7 and cleaved PARP, indicating apoptosis, with the level depending on the concentration used. The signal level in cells treated with the 5-FLU NPs was higher than in the case of free 5-FLU, suggesting stronger apoptosis activation even at a lower dose. The expression of the p53 protein also increased significantly, reflected by the accumulation of p53 in the cell nucleus. Such high p53 expression correlates with a strong stress response and the activation of apoptotic pathways in cells exposed to the NPs.

**Expression of apoptosis markers after 48 h of treatment with SiNPs-NH_2_-PO_3_-5-FLU-PEG2000**

The expression profile of apoptosis markers for SiNPs-NH_2_-PO_3_-5-FLU-PEG2000 generally coincided with observations for free 5-FLU, indicating that PEG modification of the NP does not impair the action of the drug. After 48 h of treatment with 10 µM and 25 µM of 5-FLU SiNPs and PEG-SiNPs, the expression of active caspase-7, cleaved PARP, and p53 protein in the MCF7 cells was similar to 10 µM of free 5-FLU, indicating a slight initial activation of caspase-7; however, for 25 µM of PEG-NPs, the signal intensity was significantly higher than for 25 µM of free 5-FLU.

**Caspase 7**

**PARP**

**P53**

## 3. Discussion

To the best of our knowledge, this is the first study to investigate silica nanoparticles simultaneously modified with phosphate groups and PEG 2000 chains as a platform for 5-fluorouracil delivery. The introduction of phosphate groups, in combination with PEGylation, is expected to modulate surface charge, surface-related interactions, and drug release profile, offering a distinct approach compared to previously reported silica-based systems. A previous study [57] demonstrated that the surface functionalization of mesoporous silica nanoparticles with sulfonate functional groups, which carry a strong negative charge, enabled strong interactions with doxorubicin (Dox). In the result, higher loading efficiency of Dox in anionic modified MSNs was obtained (15%), compared to results obtained for cationic modifications (with -NH_2_ groups and polyethylene imine -PEI, 0.2–0.3%). Dox possesses a positive charge (pKa = 8.3) [58], which translates into electrostatic adsorption of Dox to the negatively charged mesoporous silica nanoparticles. Based on the relationships described above, we functionalized the surface of the SiNPs with both -NH_2_ and -PO_3_ groups to increase their negative surface charge. This was done to enhance the likelihood of interactions between the nanoparticles and daunorubicin, which has a spatial structure similar to that of Dox and a comparable pKa value [59].

Silica is a common natural substance that possesses great biocompatibility with the human body, and is regarded as nontoxic and biocompatible by the U.S. Food and Drug Administration (Title 21: Sec. 172.480, 2017) [60]. As such, silica appears to offer promise as a substrate for drug delivery systems (DDSs). These systems can be used to deliver various macromolecules, such as peptide drugs, antibody–drug conjugates, and DNA- and RNA-based drugs for treating inter alia diabetes, cancer, and neurotherapeutic pain. However, the translation of DDSs from the lab to clinical scenarios is faced by a number of obstacles, such as premature drug release, resulting in intolerable adverse effects [61]. One way to overcome the limitations of conventional DDSs based on nanoparticles involves the use of surface functionalization to enable targeted therapeutic delivery [62].

Various methods can be used to create silica nanoparticles (SiNPs) for use in DDSs, such as reverse microemulsion [63], flame synthesis [64], or sol–gel [65]. Reverse microemulsion is hampered by high cost and difficulty in removing surfactants from the final products [66], while flame synthesis lacks accurate control of nanoparticle size and morphology and phase composition [67]. Therefore, a one-pot biphasic sol–gel method conducted at room temperature was used in the present study. Nevertheless, SiNPs have many desirable properties: their structure houses porous channels which offer a large internal space for drug loading and allow the drugs to gradually diffuse out according to dissolution media.

The size and surface zeta potential of the created SiNPs were determined by DLS measurements. Prior to drug loading, the nanoparticles exhibited a relatively low absolute zeta potential (−5.3 ± 0.4 mV), whereas loading with 5-fluorouracil increased the magnitude of the surface charge (−13.5 ± 1.3 mV). This increase in absolute zeta potential enhances electrostatic repulsion between particles, improves colloidal stability, and reduces aggregation, which is reflected in the decrease in average hydrodynamic diameter (from 504.7 ± 21.1 nm to 271.7 ± 16.2 nm). Nanoparticle transport is strongly influenced by its surface charge properties. When nanoparticles are immersed in an aqueous medium, they acquire a surface charge caused by protonation or deprotonation on the particle surface. The interaction between the surface charge and dissolved ions results in the formation of an electrical double layer (EDL) surrounding the charged particles [68], which directly contributes to the hydrodynamic radius measured by DLS. This measurement reflects not only the nanoparticle core but also the surrounding interfacial water layer. Previous studies [69] have shown that amorphous silica nanoparticles possess a dynamic interfacial region composed of silanol/siloxide groups and structured water, which is highly sensitive to surface charge and functionalization. Modifications in surface chemistry can therefore influence electrostatic interactions at the interface, ultimately affecting the properties of the hydration layer. Surface functional groups, such as amino and phosphate groups, can promote water structuring and expand the hydration shell, leading to larger apparent particle sizes. Literature evidence indicates that the surface charge of nanoparticles plays a key role in determining their effective size in dispersion [70]. Following drug loading, partial masking of surface groups and altered interactions with water may reduce the thickness of the hydration layer, resulting in smaller measured diameters [71].

Nanoparticle size also affects their stability, cellular uptake, and biodistribution, with smaller NPs demonstrating prolonged circulation time and accumulation at the defined tissue, as well as a lower risk of uptake by the reticuloendothelial system (RES) [72].

The size of the nanoparticles observed in SEM images (~40 nm) differs from that obtained in DLS measurements (~200 nm). Such a large discrepancy between the results is related to the differences in measurement principles of these two techniques. SEM provides information on nanoparticles in their dried state, whereas in DLS measurements, the nanoparticles were dispersed in PBS solution. As a result, the SEM data correspond to the core diameter, while DLS reflects the hydrodynamic diameter of nanoparticles in PBS. The larger sizes observed in DLS are associated with, e.g., the formation of aggregates, as described above. SEM images acquired at lower magnification further confirm the formation of aggregates, visible as a sponge-like network of interconnected particles.

The Horvath–Kawazoe analysis revealed a predominant proportion of pores with a diameter of approximately 0.8 nm, confirming the highly microporous nature of the sample. Such small pores may promote stronger adsorbent-adsorbate interactions. At the same time, the presence of ultramicropores may limit the diffusion of larger molecules into the interior of the structure. A decrease in specific surface area (BET) of SiNPs-NH_2_ and SiNPs-NH_2_-PO_3_ may indicate effective surface functionalization, leading to partial filling of the micropores and a reduction in the available adsorption surface area. This observation corresponds with the results regarding pore volume. The decrease in pore volume (BJH) can be attributed to the partial blocking and filling of the pores by the modifying layer. In the case of microporous materials, even a small amount of the deposited phase can significantly limit the accessibility of the porous structure to nitrogen.

The weak visibility of amino and phosphate groups in the FTIR spectra prior to drug loading can be attributed to their low surface coverage and the dominance of intense Si–O–Si bands, as well as band overlapping with silanol and water-related vibrations [73]. Silica has very strong bands (~1000–1100 cm^−1^) [74], which mask weak signals from organic groups, especially when the degree of functionalization is low. The surface density of -NH_2_ and -PO_3_ groups is relatively low compared to the bulk SiO_2_ matrix. Prior to drug loading, the nanoparticles tend to form aggregates, which may reduce the effective exposure of surface functional groups and promote intermolecular interactions (e.g., hydrogen bonding), resulting in band broadening and reduced spectral resolution [75]. Aggregation reduces the accessible surface area and may lead to partial shielding of surface functional groups, while simultaneously enhancing the contribution of bulk Si–O–Si vibrations. Furthermore, structural changes and interparticle interactions in aggregated systems are known to affect FTIR band intensity and shape [76]. The situation changes after drug loading. Several changes contribute to the improved visibility of these functional groups. The incorporation of 5-FLU likely enhances nanoparticle dispersion (as supported by the decrease in hydrodynamic diameter), leading to better exposure of surface moieties. In addition, interactions between 5-FLU and surface functional groups (such as hydrogen bonding or electrostatic interactions) can modify the local chemical environment, resulting in shifts and/or sharpening of the corresponding FTIR bands. Furthermore, partial disruption of interactions between -NH_2_ groups and surface silanols may also contribute to improved spectral definition.

The ability of the modified SiNPs to encapsulate and release 5-fluorouracil was evaluated using HPLC. The results indicate a relatively low degree of 5-FLU loading compared to the theoretical value. The 5-FLU molecule has an octanol–water partition coefficient of 0.89 and a pKa of 8.02, indicating a preference for polar solvents [77]. The relatively low drug loading (DL 2.4%, EE 24%) might be explained by the strong solvation of 5-FU in the aqueous phase, which limits its partitioning into the nanoparticle matrix, as well as by weak interactions with the surface of the silica-based carrier. In addition, the absence of strong hydrophobic or specific binding interactions further contributes to the limited adsorption capacity [78]. Moreover, a portion of drug molecules may remain unbound or be removed during purification and separation steps. Additionally, diffusion limitations into mesoporous structures and competitive interactions with surface functional groups may reduce loading efficiency [79].

Based on the drug loading (2.4%), the total amount of 5-fluorouracil in 32 mg of nanoparticles was approximately 0.768 mg. When dispersed in 16 mL of PBS, this corresponds to a maximum theoretical concentration of ~0.048 mg/mL. Considering that the solubility of 5-FLU in PBS is approximately 8 mg/mL [80], the achieved concentration is significantly below its saturation solubility (less than 1%). Therefore, the release process was not limited by solubility effects.

The discrepancy between the theoretical drug loading (9.1%) and the value measured by HPLC (2.4%) may arise from several physicochemical and process-related factors. First, the theoretical value assumes ideal adsorption conditions and complete utilization of all available active sites, which is not achieved in practice. In a real system, a portion of drug molecules may remain unbound or be removed during purification and separation steps. Additionally, diffusion limitations into mesoporous structures and competitive interactions with surface functional groups may reduce loading efficiency [79].

The incorporation of PEG chains into the SiNPs resulted in slower and more controlled release of 5-fluorouracil compared to non-PEG SiNPs. Such gradual drug release offers a significant advantage over conventional drug administration based on rapid intravenous injection or infusion [81]. Sustained release allows better control of systemic drug concentration, maintaining them within the therapeutic concentration range and limiting fluctuations. Notably, drug release occurs not only under physiological conditions but is even enhanced in the mildly acidic environment (pH 5.0). Extracellular tumor pH is typically in the range of 6.4–6.8, while pH 5.0 is characteristic of endosomal/lysosomal compartments [82]. This is driven by the accumulation of acidic metabolic byproducts formed by increased metabolic activity and insufficient perfusion [83]. In our study, pH 5.0 conditions are intended to mimic intracellular acidic organelles following nanoparticle internalization. Such a dependency results in the preferential release of the drug at the tumor site, with enhanced localized therapeutic efficacy. The difference in release between pH 7.4 and 5.0 for the PEG-free system appears relatively small. This behavior can be attributed to the strong hydrophilic character of 5-FLU [84] and its multiple hydrogen-bonding interactions with the silica surface, which result in a relatively diffusion-controlled release profile [85]. Additionally, within the studied pH range, the protonation state of surface amino groups changes only moderately, while phosphate groups remain largely ionized. Therefore, the overall change in surface charge density is not sufficient to induce a pronounced switch in release behavior for the non-PEGylated system.

In contrast, the acidic environment still promotes partial weakening of drug–surface interactions [86], which is reflected in the observed (although moderate) increase in release at pH 5.0. Chen et al. [35] investigated the therapeutic efficacy of fluorouracil and ß-lapachone (ARQ761 in its clinical form) encapsulated within mesoporous silica nanoparticles for the treatment of head and neck squamous cell carcinomas. Consistent with our findings, the authors demonstrated that pH-responsive drug release under acidic conditions is beneficial for anticancer therapy. Moreover, similarly to our study, the presence of PEG chains contributed to a slower and more controlled release of the drug.

Hydrophobic and/or charged nanoparticles tend to have shorter circulation half-lives [87] because they are readily opsonized. Therefore, nanoparticles intended for systemic administration are typically modified with an electrically neutral, hydrophilic surface layer, commonly referred to as a “stealth coating.” PEG2000 forms a hydrophilic steric layer on the surface of nanoparticles, which brings several advantages, such as an anti-opsonization effect and reduced recognition by immune system cells [88]. It is possible that PEGylation of SiNPs-NH_2_-PO_3_-5-FLU may improve pharmacological properties, such as circulation stability and reduced recognition by the immune system, although these features require confirmation in in vivo studies.

Continuous low-dose administration of 5-FLU has become a widely used treatment approach for pretreated metastatic breast cancer over the past few decades, facilitated by the introduction of Medtronic infusion pumps [89]. The MCF7 cell line was selected as a well-established and widely used in vitro model for evaluating cytotoxicity, apoptosis, and nanoparticle–cell interactions [90]. Its extensive characterization and frequent use in drug delivery studies enable comparison with previously reported systems and ensure good reproducibility of results. Previous reports [91,92] on silica-based nanocarriers evaluated in MCF-7 cell lines also demonstrated enhanced anticancer activity in MTT assays. In these studies, drug-loaded nanoparticles exhibited greater reduction in cell viability than the free API, suggesting improved cellular uptake and therapeutic efficiency of the nanoscale delivery system. However, the use of only the MCF-7 cell line represents a limitation; differences in drug metabolism, proliferation rate, and nanoparticle uptake between cell lines may influence the observed effects [93]. Therefore, future investigations should include additional cancer cell models, normal cell lines, as well as in vivo studies, to better assess the efficacy, selectivity, and translational potential of the proposed nanosystem.

The use of nanoparticle-based carriers for 5-FLU offers potential benefits in therapy. Our findings demonstrate that SiNPs-NH_2_-PO_3_-5-FLU nanoparticles can induce a stronger apoptotic response than free 5-FLU at comparable concentrations, particularly so at the lower dose of 10 µM; this indicates improved intracellular delivery or release of the drug.

The lower activity of PEGylated nanoparticles (10 μM) compared to non-pegylated nanoparticles may result from the so-called “stealth layer” effect [94]. It turns into the slowed cellular internalization [95] and may lead to reduced cytotoxicity compared to non-PEGylated particles, despite the potentially more controlled drug release. From a therapeutic perspective, nanoparticles also offer the possibility of accurately targeting the drug to tumor tissue and enabling more controlled drug release, which together may increase treatment effectiveness and reduce side effects.

## 4. Materials and Methods

### 4.1. Reagents and Drugs

Triethanolamine (TEA, ≥99%) (Merck, Darmstadt, Germany), cetyltrimethyl ammonium chloride (CTAC, 25 wt% in water) (Merck, Darmstadt, Germany), n-hexane (Merck, Darmstadt, Germany), tetraethyl orthosilicate (TEOS, 99%) (Merck, Darmstadt, Germany), toluene (≥99.5%) (Merck, Darmstadt, Germany), 3-aminopropyl-triethoxy silane (APTES, 99%) (Merck, Darmstadt, Germany), trihydroxysilylpropyl methyl-phosphonate (THMP, 50 wt% in H_2_O) (Merck, Darmstadt, Germany), phenol red-free DMEM, 1,1′-dioctadecyl-3,3,3′,3′-tetra-methylindocarbocyanine perchlorate (Thermo Fisher Scientific, Waltham, MA, USA), and poliethylenoglicol2000 (PEG2000) (Merck, Darmstadt, Germany). Acetonitrile ≥ 99.90% (GC), gradient-grade HPLC, and Methanol HPLC isocratic grade from (Avantor Performance Materials, Warsaw, Poland), ortho-phosphoric acid 85% (Merck, Darmstadt, Germany), tripotassium phosphate from (Witko, Łódź, Poland). Ultrapure water was obtained from Hydrolab Ultra UV (Hydrolab, Straszyn, Poland). 5-Fluorouracil was obtained from Cayman Chemical Company (Ann Arbor, MI, USA). Phosphate-buffered saline (PBS) at pH 5.0 and pH 7.4 and 1.5 mM K_3_PO_4_ buffer were prepared.

### 4.2. Synthesis of Silica Nanoparticle Platforms (SiNPs)

**Figure 11:**
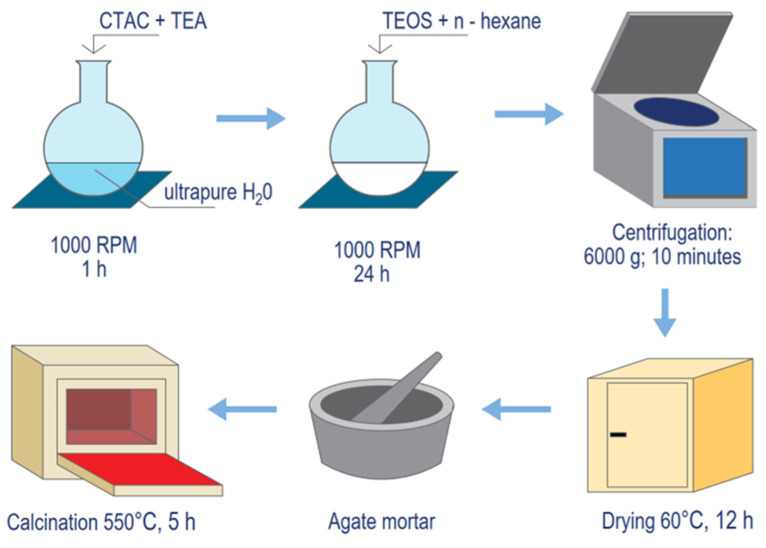
Graphical presentation of the synthesis.

Firstly, 1 mL of CTAC and 0.54 mL of TEA were introduced into a round-bottom flask containing 18 mL of ultrapure water on a magnetic stirrer, and were stirred at 1000 rpm for one hour. Following this, 2 mL of TEOS and 18 mL of n-hexane were added to the reaction vessel and the solution was stirred for 24 h. Silica nanoparticles (SiNPs) were obtained by centrifuging at 6000 RPM for 10 min. After the supernatant was decanted, the precipitate was rinsed three times with 70% ethanol to remove unreacted substances. The SiNPs were dried in a porcelain crucible at 60 °C for 12 h and then crushed with an agate mortar to obtain a fine white powder. Finally, residual surfactants were removed by calcining the SiNPs in a muffle furnace at 550 °C for five hours. A schematic illustration of the synthesis is presented in Figure 11.

### 4.3. Surface Modifications of SiNPs

Amino and phosphate groups were covalently bonded to the SiNPs’ surface. The first step was amine functionalization (SiNPs-NH_2_).

Briefly, after calcination, 500 mg of SiNPs was added to a round-bottom flask with 70 mL of toluene and stirred at 50 °C in a sealed environment. After 30 min, 500 μL of APTES was added and the whole mixture was stirred at 115 °C for 18 h, underneath an air cooler to prevent the permeation of moisture. The suspension was then centrifuged (6000 RPM for 10 min) and washed three times with 70% ethanol. SiNPs-NH_2_ were obtained by drying at 60 °C for 12 h and then crushed into a fine powder.

Following this, the surface of the SiNPs was modified with phosphate groups (SiNPs-NH_2_-PO_3_). Briefly, 0.5 mL of THMP was mixed with 50 mL of ultrapure water. The pH of the solution was adjusted to pH 5.0 by the addition of hydrochloric acid (HCl). Next, 500 mg SiNPs-NH_2_ was introduced to the mixture, which was sonicated for 15 min to disrupt SiNP agglomerates. The suspension was stirred at 100 °C for 18 h (1000 rpm) under an air condenser. The SiNPs-NH_2_-PO_3_ were obtained by centrifugation (6000 RPM, 10 min) and washed with 70% ethanol three times. They were then dried at 60 °C for 12 h and crushed into a fine powder.

### 4.4. Drug Loading on SiNPs-NH_2_-PO_3_

The drug (5-FLU) was loaded into SiNPs-NH_2_-PO_3_ by solvent evaporation [96]. Briefly, 8 mL of 14 mM aqueous 5-FLU solution was prepared. Of this, 1 mL was collected for further HPLC analysis, and 100 mg of SiNPs-NH_2_-PO_3_ was dispersed in a drug solution (14 mM aqueous 5-FLU). The suspension was sonicated for five minutes and then stirred at 500 RPM for two hours. The solvent was then evaporated using a rotary evaporator with a water bath. Following this, a volume of water equivalent to that of the drug encapsulation (5-FLU) solution was added to the flask containing the nanoparticles to dissolve any unencapsulated 5-FLU. The SiNPs were obtained by centrifugation (6000 RPM, 10 min) and vacuum-dried in a desiccator. The supernatant was analyzed by HPLC to determine the amount of unencapsulated 5-FLU.

### 4.5. PEG2000 Conjugation with SiNPs-NH_2_-PO_3_-5-FLU

**Figure 12:**
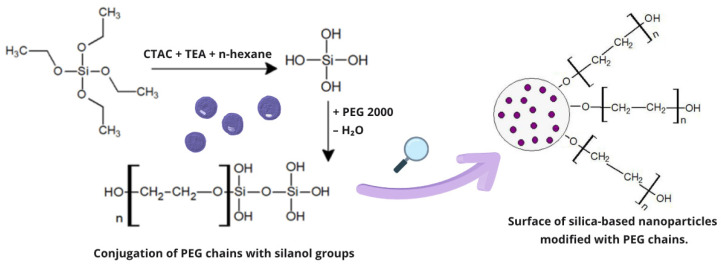
Schematic illustration of the functionalization of silica nanoparticle surfaces via attachment of polyethylene glycol (PEG) chains to silanol groups.

The SiNPs-NH_2_-PO_3_-5-FLU (30 mg) obtained by solvent evaporation were added to 2 mL of ultrapure water. In a separate vessel, 45 mg of PEG 2000 was dissolved in 3 mL of 1.5% acetic acid. The PEG 2000 solution was added dropwise to the nanoparticle suspension with a burette for one hour, while stirring continuously. The SiNPs were isolated by centrifugation (6000 RPM, 10 min) and then dried under vacuum in a desiccator. The PEG2000 conjugation was carried out at room temperature and on ice. A schematic illustration of the conjugation is presented in Figure 12.

### 4.6. Scanning Electron Microscope (SEM)

The morphological characteristics of SiNPs-NH_2_-PO_3_-5-FLU and SiNPs-NH_2_-PO_3_-5-FLU-PEG2000 were determined using scanning electron microscopy (SEM) (Carl Zeiss, Oberkochen, Germany). Images were captured using a Zeiss Ultra Plus at an acceleration voltage of 8 kV. The sample was placed onto a conductive carbon tape. Excess powder was removed. The sample was then transferred to a vacuum sputter coater (Quorum model Q150TES), where a thin (approximately 5 nm) layer of an Au60%Pd40% alloy was deposited via plasma sputtering.

### 4.7. Dynamic Light Scattering (DLS) and Zeta Potential (ZP)

The sizes of the SiNP particles and surface-modified SiNPs (SM-SiNPs) were assessed by DLS. The SiNPs/SM-SiNPs were suspended in 1 mg/mL PBS (pH 7.4) and sonicated for 10 min. The suspensions were diluted to 10 μg/mL and 1 μg/mL in PBS at pH 7.4 in disposable folded capillary zeta cells, and then taken for SiNP size and zeta potential measurement.

### 4.8. Braunauer–Emmet–Teller (BET), Barrett–Joyner–Halenda (BJH), and Horvath–Kawazoe (HK) Pore Size Analysis

The specific surface area measurement was carried out using BET analysis. The pore volume was evaluated using the BJH method applied to the adsorption/desorption branches of the isotherm. Horvath–Kawazoe (HK) pore size analysis method was used to measure the pore size distribution. All the results were obtained using a Micromeritics 3Flex Adsorption Analyzer. Between 60 and 70 mg of nanoparticle powder was dried in the following desorption station: Smart VacPrep, Micromeritics Instrument Corporation, in vacuum. Desorption temperature: 200 °C, desorption time: 4 h. Liquid nitrogen with the following temperature was used for measurement: −195.8 +/− 0.2 °C.

### 4.9. Fourier Transform Infrared Spectroscopy (FTIR)

The functional groups on the SiNPs were analyzed by Fourier Transform Infrared Spectroscopy (FTIR) using the FTIR-ATR spectrometer (PerkinElmer, Waltham, MA, USA), frequency 400–4000 cm^−1^.

### 4.10. High-Performance Liquid Chromatography (HPLC) Analysis—Drug Loading

A calibration curve of 5-FLU was prepared in water for the concentration range 0.01 mg/mL to 1 mg/mL in (R2~0.99).

The concentration of 5-FLU in the drug solution was determined prior to encapsulation and afterwards: after drug loading, the NPs were centrifuged and the drug level in the supernatant was measured. The concentration difference between the two solutions, i.e., before and after loading, was used to calculate the amount of API encapsulated within the nanoparticles [97]. The mass of the drug loaded on the NPs was determined using a LaChrom HPLC system (Merck-Hitachi). The stationary phase used a symmetry Waters C18 (Milford, MA, USA), (200 mm × 4.6 mm × 5 μm) analytical column. The mobile phase consisted of 1.5 mM K_3_PO_4_ buffer adjusted to pH 5.0 by orthophosphoric acid and acetonitrile (95:5; *v*:*v*). The flow rate was 1.0 mL/min and the eluate was monitored at 210 nm. The injected volume was 10 μL [98].

### 4.11. High-Performance Liquid Chromatography (HPLC) Analysis—Release Study

A calibration curve of 0.01 mg/mL to 1 mg/mL 5-FLU was prepared in water (R2~0.99). The amount of 5-FLU released from the 5-FLU-SiNPs (SiNPs-NH_2_-PO_3_-5-FLU) and PEGylated SiNPs (SiNPs-NH_2_-PO_3_-5-FLU-PEG2000) was assessed using a LaChrom HPLC (Merck-Hitachi) as described previously [35].

To measure in vitro drug release, 32 mg of the 5-FLU-SiNPs or the PEGylated SiNPs containing a known amount of 5-FLU was suspended in 16 mL of PBS (pH 7.4 or 5.0). A release assay was conducted at 37 °C under continuous stirring using a magnetic stirrer (300 RPM) to ensure homogeneous mixing throughout the experiment. At a predetermined time, 1 mL of the sample was taken and centrifuged (6000 RPM, 10 min). Additionally, sink conditions were maintained. The supernatant was then analyzed by HPLC.

### 4.12. Cell Culture

MCF7 (ATCC^®^ HTB-22™) breast cancer cells were maintained in 10 mL of complete DMEM medium supplemented with 10% FBS, 50 U/mL penicillin, and 50 µg/mL streptomycin (37 °C in 5% CO_2_) in 100 mm dishes. The medium was changed two to three times per week.

### 4.13. Cell Viability Test MTT

The cytotoxicity of the SiNPs and 5-FLU on MCF7 cells was determined by the MTT (methyl thiazolyl tetrazolium) method according to Carmichael [99]. Briefly, the yellow tetrazolium bromide MTT solution is converted to purple formazan derivatives in live cells through the activity of mitochondrial dehydrogenases. The cells were cultured in 6-well plates at a density of 0.3 × 10^6^ cells/well. When the cells reached about 70% confluence, the culture media were removed. The wells were washed with PBS, and fresh DMEM media containing the test substances were added, viz. 5-FLU, SiNPs-NH_2_-PO_3_-5-FLU, or SiNPs-NH_2_-PO_3_-5-FLU-PEG2000, at 10 µM and 25 µM. The cells were then incubated for 48 h (37 °C, 5% CO_2_, pH~7.4). Following this, the medium was removed and the plates were washed twice with prewarmed PBS. The cells were then incubated at 37 °C for one hour with 1.0 mL MTT dissolved in PBS (0.5 mg/mL) per well. The MTT was removed. Any formazane derivatives were dissolved in DMSO (1.0 mL per well) and quantified by reading the absorbance at 570 nm in a spectrophotometer. Cell viability was calculated as a percentage of control values, i.e., untreated cells. Each experimental variant was performed in triplicate. All experiments were performed in triplicate and repeated independently three times. Results are presented as mean ± standard deviation (SD). Statistical analysis was performed using one-way ANOVA, and values of *p* ≤ 0.05 were considered statistically significant.

### 4.14. Immunofluorescence Analysis

The cells were cultured on a black-walled 96-well plate (0.01 × 10^6^ cells/well). After 24 h, the culture media was removed, the plate was washed with PBS, and 100 µL media containing the test substances (5-FLU, SiNPs-NH_2_-PO_3_-5-FLU, or SiNPs-NH_2_-PO_3_-5-FLU-PEG2000) at 10 µM and 25 µM were added. After 24 h, the media were removed and the cells were fixed with 3.7% formaldehyde solution at room temperature for 10 min. The plate was then washed once with 100 µL/well PBS.

Following this, 0.1% Triton X-100 solution was added and the cells were left for 10 min to permeabilize. The plate was then washed twice with PBS and blocked with 3% FBS at room temperature for 30 min. The FBS was removed, and 50 µL of the primary antibody (1:50) diluted in 3% FBS was added. The plate was incubated for one hour at room temperature, following which it was washed three times with PBS. After washing, 50 µL of the secondary antibody (dilution 1:1000) was added to each well, and the plate was left for one hour in the dark. The secondary antibody solution was removed and the plate was washed three times with PBS. The wells were then filled with 100 µL of PBS containing 2 µg/mL Hoechst 33342 to stain the nuclei. The plate was visualized using a BD Pathway 855 Bioimaging system (Beckton Dickinson).

## 5. Conclusions

The design of novel drug delivery systems, such as nanoparticle carriers for 5-FLU, represents a promising foundation for therapeutic innovation and technological integration. Our findings indicate that SiNPs-NH_2_-PO_4_-5-FLU nanoparticles can induce a stronger apoptotic response than free 5-FLU at comparable concentrations, particularly at the lower dose of 10 µM, suggesting better drug delivery to the target cells. The PEGylated SiNPs-NH_2_-PO_3_-5-FLU-PEG2000, containing PEG 2000, demonstrate similar efficacy as the free drug while potentially offering better pharmacological properties, such as circulatory stability and reduced recognition by the immune system; however, these characteristics require further in vivo study. The nanoparticles also offer additional therapeutic advantages, such as better targeting against tumor tissue and more controlled drug release, which together may increase treatment efficacy and reduce side effects.

## Figures and Tables

**Table 1:** Mean size and zeta potential of nanoparticles synthesized at room temperature.

Sample	Room Temperature	
	Mean Size [nm]	Zeta-Potential [mV]
SiNPs	261.3 ± 10.7	−5.3 ± 0.4
SiNPs-NH_2_	626.3 ± 8.2	−6.5 ± 0.8
SiNPs-NH_2_-PO_3_	504.7 ± 21.1	−12.5 ± 0.1
SiNPs-NH_2-_PO_3_-5-FLU	271.7 ± 16.2	−13.5 ± 1.3
SiNPs-NH_2_-PO_3_-5-FLU-PEG2000	282.2 ± 18.2	−12.3 ± 0.7

**Table 2:** Physical characterization of silica nanoparticles. Surface area and porosity of SiNPs before and after surface functionalization. Data are presented as mean ± SD (*n* = 3 independent experiments). Values of *p* ≤ 0.05 were considered statistically significant (one-way ANOVA).

Sample	BET Surface Area [m^2^ g^−1^]	HK Pore Size [nm]	BJH Pore Volume [cm^3^ g^−1^]
SiNPs	788	0.79	2.15
SiNPs-NH_2_	327	0.83	1.24
SiNPs-NH_2_-PO_3_	280	0.85	1.32

**Table 3:** Drug loaded, encapsulation efficiency, drug loading, and theoretical drug loading values determined by the HPLC method.

*Drug Loaded* [mg]	Encapsulation Efficiency (%)	*Drug Loading* (%)	Theoretical Drug Loading (%)
2.4	24	2.4	9.1

## Data Availability

The original contributions presented in this study are included in the article. Further inquiries can be directed to the corresponding author.

## Abbreviations

ACN	Acetonitryl
APTES	3-aminopropyl-triethoxy silane
BS	Bovine serum
CA	Citric acid
CTAC	Cetyltrimethylammonium chloride
DLS	Dynamic light scattering
DMEM	Dulbecco’s modified Eagle medium
DMSO	Dimethyl sulfoxide
FBS	Fetal bovine serum
5-FLU	5-Fluorouracil
FTIR	Fourier transform infrared spectroscopy
HPLC	High-performance liquid chromatography
MCF-7	Michigan Cancer Foundation-7
MTT	Methyl thiazolyl tetrazolium salt
PARP	Poly(ADP-ribose) polymerase
PBS	Phosphate-buffered saline
PEG2000	Polyethylene glycol 2000
PEG4000	Polyethylene glycol 4000
P53	Tumor suppressor protein
RPM	Revolutions per minute
SiNPs	Silica nanoparticles
SM-SiNPs	Surface-modified silica nanoparticles
TEA	Triethanolamine
TEOS	Tetraethyl orthosilicate
THMP	Trihydroxysilylpropyl methyl-phosphonate
SLS	Sodium lauryl sulphate
